# Commentary: the role of cytologic analysis of voided urine in the work-up of asymptomatic microhematuria

**DOI:** 10.1186/1471-2490-9-13

**Published:** 2009-09-10

**Authors:** Deep Trivedi, Edward M Messing

**Affiliations:** 1 Department of Urology, University of Rochester, School of Medicine and Dentistry, Rochester, NY 14642, USA

## Abstract

Microscopic hematuria is a common finding in patients presenting to both primary care doctors as well as urologists. Sources of microscopic hematuria include infection, stones, inflammatory disorders as well as cancer of the genitourinary tract, particularly urothelial cancer. A primary focus in the urologic workup of hematuria is to rule out cancer. This is done using radiographic studies as well as procedures such as cystoscopy and bladder biopsy. As the authors state in their article titled "The utility of serial urinary cytology in the initial evaluation of the patient with microscopic hematuria", cytologic analysis of voided urine, though attractive due to its noninvasive nature, has been found to have the neither the sensitivity, cost-effectiveness, nor the ease of administration necessary to replace more invasive diagnostics in the evaluation of microscopic hematuria.

## Commentary

The use of urine cytology to detect bladder cancer in patients who present with microhematuria has been frequently reported. This is, in no small part, due to the intuitive attractiveness of a non-invasive, relatively inexpensive screening test for a lethal disease that can be obtained in the office or clinic setting without exposing the patient to the risks of invasive procedures, surgery, radiation or contrast exposure. Another theoretical advantage of urine cytology screening is the ability to rule out malignancy, not only reducing patient concern, but also promoting cost savings through the avoidance of invasive and radiological procedures.

Unfortunately, the diagnostic capabilities of voided urine cytology are rather disappointing. Although the test has a high overall specificity (reported as up to 98%,)[1] its utility as a primary detection tool is quite marginal, because of its low overall sensitivity in detecting urothelial cancer (ranging from 40% to 76%)[2] as compared to over 91% for cystoscopy [3]. This disparity is especially pronounced in the detection of low grade tumors, with the sensitivity of voided cytology dropping to 11.1%[4]. The low sensitivity of this test is why most new urine marker studies (BTA, NMP22, Bladderchek, FISH, etc) compare their performance to that of voided urine cytology instead of cystoscopy---they will always appear more effective.

In their paper, "Utility of Serial Urinary Cytology in the Initial Evaluation of the Patient with Microscopic Hematuria" published recently in BMC Urology [5], Rosser and colleagues confirm the low sensitivity of urinary cytology at 33% (88% if atypia is considered positive) and the high specificity of urine cytology ranging from 67% (47/47+23) if atypia is considered positive to 100% (if atypia if considered negative)[5]. Furthermore, the high sensitivity of cystoscopy for the detection of bladder tumors is confirmed at 100% in this study.

Although urine cytology is relatively sensitive in detecting high grade cancer and carcinoma in situ (sensitivity 80-90%)[6], 60% of urothelial tumors at presentation are low grade and stage lesions[7]. Because of its low sensitivity for the latter cancers, voided urine cytology cannot be used to decide which patient with microhematuria can safely forgo cystoscopy. The incremental benefit of voided urine cytology to the sensitivity of cystoscopy is minimal, with the majority of its value derived from being able to detect occult carcinoma-in-situ (CIS), which represents de novo disease in only 3% of UC cases [8]. Although the authors acknowledge that no cases of urothelial CIS were present in their sample, they make no mention of the sizes or grades of the cancers detected in the 17 patients in whom biopsy-proven bladder cancer was diagnosed. If high-grade cancers were largely missed by voided urine cytology, the value of this test would be further diminished.

But perhaps the greatest problem with urine cytology not discussed in the paper is that results are quite operator dependent with the skill of the cytologist and cytologic technician being very important [9]. Also, the authors fail to elaborate on the technical aspects of cytopathologic evaluation of the urine specimens in their study (including fixation methods and time between collection and fixation/preparation) as well as the experience and number of cytopathologists who participated.

Even from an economic perspective, previously published reports suggest that voided urinary cytology is not a cost-effective test. In a series by Hofland and Mariani of 1000 consecutive patients presenting with either documented gross or microhematuria, 660 patients underwent cytologic analysis of voided urine samples as part of a work-up involving cystoscopy +/-biopsy, urography (excretory or retrograde pyelography, but excluding CT), renal ultrasound, and urine culture. Seventy-one patients in this series were diagnosed with UC. Voided urine cytology was found to be positive in 25 of the 660 patients (3.8%). Using 2002 Medicare data, the authors noted the unit cost for testing a voided urine cytology sample of $50.71 to be significantly less than that of cystoscopy ($216.54) or IVP (Intravenous Pyelogram) ($93.02). Consequently, they calculated the total cost of voided urine cytology to be $33,467 (660 × $50.71) compared to $206,442 (956 × $216.54) for cystoscopy and $89, 836 (966 × 93.02) for IVP. Urine cytology, however, was diagnostic for a life threatening condition (genitourinary malignancy, DIC, glomerulopathy) in only 21 cases (3.3%), as compared to 68 cases (7.1%) and 53 cases (5.5%) for cystoscopy and excretory urography, respectively. Furthermore, only in 4 cases (0.6%) did urine cytology provide unique information, not obtained from any other diagnostic procedure that resulted in a diagnosis of UC as compared to 64 cases and 16 cases for cystoscopy and IVP, respectively. Accordingly, the authors found the cost to produce unique, diagnostically relevant information from voided urine cytology to be $33,467/4 ($8367.00), almost double that of IVP at $5616.00 ($89,836/16) and more than double that of cystoscopy at $3235.00 ($206,442/64) [10]. Hence, despite being relatively inexpensive on a per-test basis, voided urine cytology does not appear to be a cost-effective test.

Additionally, in the workup of hematuria, microscopic or otherwise, the urologist's job goes beyond ruling out the presence or absence of urinary tract malignancy. Stones, vascular malformations, inflammatory as well as infectious lesions detectable by cystoscopy, may all result in blood in the urine. Furthermore, bladder wash cytology, which has increased sensitivity compared to cytology on voided urine specimens [11], may be obtained during cystoscopy, serving to add diagnostic value over voided urine cytology even in the face of CIS missed cystoscopically or radiologically. In sum, voided urine cytology does not obviate the need for cystoscopy. For similar reasons, voided urine cytology cannot replace radiographic studies, which can detect not only benign lesions but neoplasms outside the collecting system, for example, in the renal parenchyma.

Voided urine cytology does not appear to have a primary role in the evaluation of asymptomatic microhematuria but may have a role as a supplement to cystoscopy and excretory urography in a minority of cases (for example when "invisible" diseases such as CIS are suspected.) However, hematuria is a "late" detector of bladder cancer and almost never is voided urine cytology the single test during a hematuria workup that detects urothelial cancer [12].

One may argue that in the above series by Hofland and Mariani, for the 4 patients in whom malignancy was solely detected by urine cytology that this test proved to be very valuable. But the question arises, "for a disease with a rather low prevalence (urothelial CIS), is a test with a very small unique diagnostic yield justified?" If one extrapolates the cost analysis by Hofland and Mariani to the general population, the magnitude of inefficiency becomes glaringly obvious. Asymptomatic microhematuria is prevalent in up to 21% of the US population [13] or roughly 60 million Americans. If each individual was only tested once, cytology alone would cost over $3 billion. Furthermore, if a similar yield of diagnostically unique information of 0.6% was applied to this population, over $2.98 billion would be spent on a relatively insensitive and redundant test. In today's political environment where increased pressure is being placed on the health care system to reign in costs, the elimination of voided urinary cytology in the workup of asymptomatic microhematuria has the potential to effect significant savings.

Perhaps with its high documented positive predictive value (assuming atypia is not considered positive), positive urine cytology can be used to direct high risk patients to operating room procedures, obviating the need for office cystoscopy since a biopsy and/or ureteroscopy would be needed as a supplement to surveillance cystoscopy. Furthermore, there may be a role in using voided urine cytology in patients with a history of bladder cancer who are at risk for recurrence and disease progression.

With the availability of numerous other more sensitive urine based assays to detect urothelial cancer, the role for voided urine cytology in the diagnosis of urinary tract malignancy (other than CIS) appears to be diminishing. The search for a sensitive, non-invasive, inexpensive, readily available test for urothelial cancer must go on.

## Competing interests

The authors declare that they have no competing interests.

## Pre-publication history

The pre-publication history for this paper can be accessed here:



## References

[B1] Castro G, Fernandez F, Corriente M (2008). "Usefulness of urine cytology in the diagnosis of bladder cancer: relationship with pathologic findings". Acas Urol Esp.

[B2] Grossfeld GD, Litwin MS, Wolf JS, Hricak H, Schuler CL, Agerter DC, Carroll PR (2001). "Evaluation of asymptomatic microscopic hematuria in adults: the American Urological Association Best Practice Policy-Part II: patient evaluation, cytology, voided markers, imaging, cystoscopy, nephrology evaluation and followup.". Urology.

[B3] Grossman HB, Soloway M, Messing E, Katz G, Stein B, Kassabian V, Shen Y (2006). "Surveillance for recurrent bladder cancer using a point-of-care proteomic assay". JAMA.

[B4] Kumar A, Kumar R, Gupta NP (2006). "Comparison of NMP22 BladderChek test and urine cytology for the detection of bladder cancer.". Jpn J Clin Oncol.

[B5] Nakamura K, Kasraeian A, Iczkowski KA, Chang M, Pendleton J, Anai S, Rosser CJ (2009). "Utility of Serial Urinary Cytology in the Initial Evaluation of the Patient with Microscopic Hematuria.". BMC Urology.

[B6] Brown F (2000). "Urine Cytology-Is it still the Gold Standard for Screening?". Urol Clinics of North America.

[B7] Messing EM, Young TB, Hunt VB (1995). Comparison of bladder cancer outcome in men undergoing hematuria home screening versus those with standard clinical presentations. Urology.

[B8] Nese N, Gupta R, Bui MH, Amin MB (2009). "Carcinoma in situ of the urinary bladder: review of clinicopathologic characteristics with an emphasis on aspects related to molecular diagnostic techniques and prognosis.". J Natl Compr Canc Netw.

[B9] Raitanen MP, Aine R, Rintala E, Kallio J, Rajala P, Juusela H, Tammela TL, FinnBladder Group (2002). "Differences between local and review urinary cytology in diagnosis of bladder cancer. An interobserver multicenter analysis.". Eur Urol.

[B10] Hofland CA, Marianai AJ (2004). "Is urine cytology required for a hematuria evaluation.". J Uro.

[B11] Badalament RA, Hermansen DK, Kimmel M, Gay H, Herr HW, Fair WR, Whitmore WF, Melamed MR (1987). "The sensitivity of bladder wash flow cytometry, bladder wash cytology, and voided cytology in the detection of bladder carcinoma.". Cancer.

[B12] Madeb R, Messing EM (2008). "Long-term outcome of home dipstick testing for hematuria.". World J Urol.

[B13] Grossfeld GD, Wolf JS, Litwan MS, Hricak H, Shuler CL, Agerter DC (2001). Asymptomatic microscopic hematuria in adults: summary of the AUA best practice policy recommendations. Am Fam Physician.

